# Elucidating the Reaction Pathways in the Synthesis of Organolead Trihalide Perovskite for High-Performance Solar Cells

**DOI:** 10.1038/srep10557

**Published:** 2015-05-28

**Authors:** Baohua Wang, King Young Wong, Xudong Xiao, Tao Chen

**Affiliations:** 1 Department of Physics, The Chinese University of Hong Kong, Shatin, N. T., Hong Kong, China

## Abstract

The past two years have witnessed unprecedentedly rapid development of organic–inorganic halide perovskite–based solar cells. The solution–processability and high efficiency make this technology extraordinarily attractive. The intensive investigations have accumulated rich experiences in the perovskite fabrication; while the mechanism of the chemical synthesis still remains unresolved. Here, we set up the chemical equation of the synthesis and elucidate the reactions from both thermodynamic and kinetic perspectives. Our study shows that gaseous products thermodynamically favour the reaction, while the activation energy and “collision” probability synergistically determine the reaction rate. These understandings enable us to finely tune the crystal size for high-quality perovskite film, leading to a record fill factor among similar device structures in the literature. This investigation provides a general strategy to explore the mechanism of perovskite synthesis and benefits the fabrication of high–efficiency perovskite photoactive layer.

The recent exploitation of organic–inorganic hybrid perovskite in solar energy conversion arouses new academic curiosity^1^,^2^, which is mainly stimulated by the achievable power conversion efficiency (PCE) exceeding 20%^3^,^4^, comparable to the conventional vacuum deposited thin film solar cells based on Si (21.2%), CIGS (20.8%) and CdTe (20.4%)^4^. The pioneering work utilizing methylammonium lead halide perovskite in dye sensitized solar cells showed a PCE of 3.8% and was further improved to 6.5%^5^,^6^, while both of them suffered from a common problem that the perovskite degraded easily in the liquid electrolyte. The stability was considerably improved after using solid hole transport material and the efficiency was boosted exceeding 9%^7^. In addition to the high PCE, another attractive characteristic of perovskite solar cell is the feasibility in solution-processed fabrication, which offers a cost–effective printable strategy for large–area device fabrication^8^,^9^. The “solution processing” usually begins with coating the precursor mixture on a substrate, followed by annealing the precursor film at an elevated temperature to evaporate the solvent, initiate the chemical reaction towards perovskite and facilitate the crystallization and film formation. This fabrication involves complicated solid-state reaction and crystallization procedures.

Typically, the perovskite adopts a chemical formula denoted by *AMX*_3_, where *A* is methylamine, *M* is metal element and *X* represents halide element^10^. The materials that meet the electrical and optical requirements for high–efficiency photovoltaic devices are organolead or organotin iodide based perovskites, with bromine or chlorine doping in some cases^11^,^12^,^13^,^14^. The chemical synthesis can be classified into two categories. One is via the reaction between MCl_2_ (M = Pb or Sn) and CH_3_NH_3_X (X = Br or I) with a molar ratio of 1:3. The preparation of CH_3_NH_3_PbI_3-*x*_Cl_*x*_ film through reaction (1) is a typical example^15^. The other category of synthesis is by means of the reaction between MX_2_ (M = Pb or Sn, X = Br or I) and CH_3_NH_3_X (X = Br or I) with a molar ratio of 1:1, such as the reaction between PbI_2_ and CH_3_NH_3_I for the synthesis of CH_3_NH_3_PbI_3_ (reaction 2)^7^.


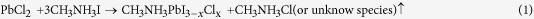



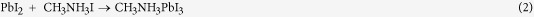


The reported high–efficiency devices are mostly based on these two reactions^16^,^17^. We thus choose them as model systems to probe the reaction mechanism. The past investigations have gained several lines of empirical evidences regarding the materials synthesis. First, the barriers for both of the reactions are quite low, enabling reaction at mild conditions, while the exact activation energies required for the reactions are unresolved. Second, in addition to the main product, the side products are unspecified, especially for reaction (1), which brings about ambiguity of the reaction mechanisms and impedes further improvement of the film quality for high-efficiency devices. Third, reactions (1) and (2) result in different film morphologies. Reaction (1) can easily lead to uniform films on a planar substrate, while reaction (2) usually generates branchlike crystals on a planar substrate in case the mesoporous scaffold is absent. This morphological difference is associated with chemical reaction, crystallization and film formation nature that are still remained undiscovered. Here, we first identify the products of the reaction and establish the chemical equations. Afterwards, we analyze the reaction from both thermodynamic and kinetic perspectives and discover their impacts on film formation behavior. With these understandings, a method to precisely control the crystal size domain for optimal device performance is developed, which ultimately leads to an improvement of the device efficiency by 22.3%.

## Results and Discussion

**Figure 1:**
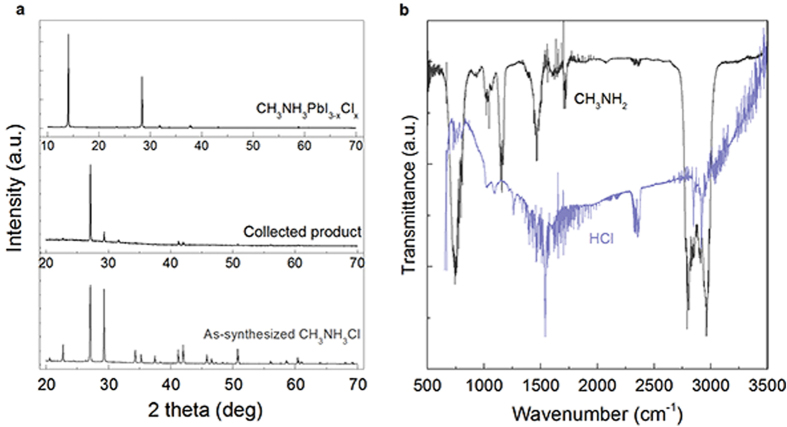
Structure characterization of the products associated with reaction in the PbCl_2_ + 3CH_3_NH_3_I system. **a**, XRD patterns of the final perovskite synthesized at 100 ^o^C for 45 min, the solidified gaseous substance collected from the PbCl_2_ + 3CH_3_NH_3_I reaction system, and the as-synthesized CH_3_NH_3_Cl for comparison. **b**, FTIR spectra of gaseous product generated from the PbCl_2_ + 3CH_3_NH_3_I reaction system.

**Figure 2:**
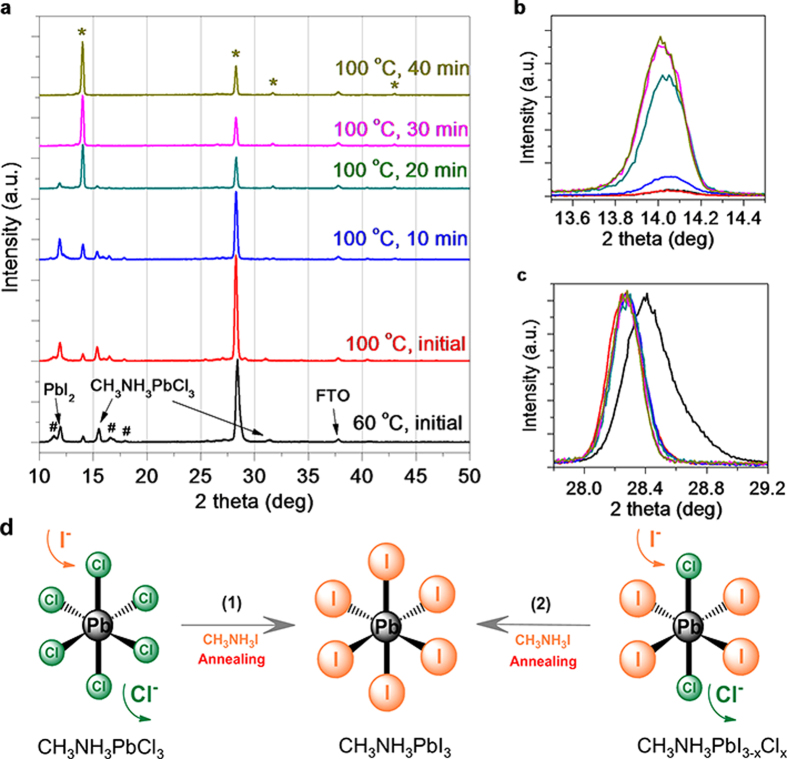
XRD characterization. **a**, *in situ* XRD monitoring of the precursor film (PbCl_2_ + 3CH_3_NH_3_I system) after annealing at 60 ^o^C for 20 min for pre-drying, and 100 ^o^C up to 40 min, in which *#* indicates the unknown peaks and * indicates the diffractions from perovskite (CH_3_NH_3_PbI_3_). The XRD patterns are offset for easy observation. **b**, Enlarged (100) diffraction peak as in Fig. 2a. **c**, Normalized (200) diffraction peak as in Fig. 2a. **d**, Schematic illustration of the replacement of Cl^−^ in the Cl-rich perovskite by I^−^ for the formation of Cl-poor perovskite (the CH_3_NH_3_ ion at the corner of the cube is omitted), where the thermodynamically favourable process would finally lead to the formation of all-iodide perovskite CH_3_NH_3_PbI_3_.

To fabricate CH_3_NH_3_PbI_3−*x*_Cl_*x*_, a precursor solution containing PbCl_2_ and CH_3_NH_3_I with a molar ratio of 1:3 in dimethylformamide (DMF) was prepared (donated as “PbCl_2_ + 3CH_3_NH_3_I system”). The mixed solution was then spin–coated on TiO_2_ compact layer on an FTO–coated glass. The formation of final perovskite film was achieved by annealing at 100 ^o^C for 45 min. X-ray diffraction (XRD) pattern shows typical (110) and (220) peaks centered at 14.1^o^ and 28.4^o^ (Fig. 1a)^3^,^18^. According to energy-dispersive X-ray spectroscopy (EDS, Supplementary Table 1), the chlorine content was found to be smaller than 2%, and sometimes undetectable. This indicates a very small value of *x* in the end product. It is usually suspected that the formation and subsequent sublimation (or decomposition) of CH_3_NH_3_Cl in the film accounts for negligible chlorine left in the final perovskite^19^,^20^. However, there has been no evidence for this assumption. Herein, we attempt to specify the gaseous product of the reaction. By analyzing the reaction given by (1), the gaseous products could be acidic HCl, HI, basic NH_3_, CH_3_NH_2_ or neutral CH_3_Cl, etc. To obtain a definite answer, we managed to separately collect the acidic or basic gases by filtering through acidic or basic media. Fourier transform infrared spectroscopy (FTIR) is used to characterize the vibrational modes of the collected gases in a gas cell. When the gas product was purged through NaOH powder, the spectrum (Fig. 1b) shows peaks at 2800-2961, 1467, 1140 and 750 cm^−1^, which are in agreement with the characteristic CH_3_ stretching, CH_3_ deformation, CH_3_ wagging and NH_2_ wagging vibrational modes of CH_3_NH_2_, respectively^21^. The standard spectrum from literature is provided in the Supplementary Fig. 1. Alternatively, the reaction product was filtered through P_2_O_5_ to collect the acidic gaseous products. FTIR measurement shows fingerprint similar to vibrational modes of HCl especially in the range 2840-3145 cm^−1^ (Fig. 1b and Supplementary Fig. 1). To confirm the identity of the reaction products, we conducted a test by annealing the film in an atmosphere containing either HCl or CH_3_NH_2_. Depending on the gas pressure of HCl or CH_3_NH_2_, the reaction speed can be retarded by certain extends. When the precursor film is annealed in an atmosphere containing NH_3_, the process is greatly accelerated and the reaction completes within 2 min (Supplementary Fig. 2). It is ascribed to the quick removal of HCl by combining with NH_3_. According to Le Chatelier’s principle, both HCl and CH_3_NH_2_ serve as the equilibrium shifting factors that resist the forward reaction. Therefore, in combination with the FTIR analysis, the volatile products are identified as HCl and CH_3_NH_2_. To inspect whether there is CH_3_NH_3_Cl sublimated, we tried to collect the product by covering a glass slide onto the precursor film. Upon heating, the volatile species can be deposited onto the upper slide. As a result, the XRD pattern of the collected substance matches that of the as-synthesized CH_3_NH_3_Cl (Fig. 1a). However, both our investigation (Fig. 2a) and literaturereport did not show diffraction peak of CH_3_NH_3_Cl during the reaction^3^,^15^,^20^. Therefore, the as-collected CH_3_NH_3_Cl is a product of the reaction between escaped HCl and CH_3_NH_2_.

Because of the generation of gaseous products, we also observed that if the reaction was conducted at a reduced pressure, the reaction would speed up because of the fast removal of the gaseous products. This is a typical characteristic of the solid-state reaction involving gaseous product generation. On the other hand, the reaction rate is greatly decreased when the reaction was performed in an enclosed system where the generated gas is retained in the system, indicating that the escape of gaseous product is a driving force for the reaction. To examine the reversibility of the reaction, we place a perovskite film facing a precursor film. The bottom precursor film was then heated up to 100 ^o^C. The rationality behind this experiment is that HCl and CH_3_NH_2_ generated from the precursor film are able to enter into the upper pre-formed perovskite film and react with it. As a consequence, the surface of the perovskite film was observed to turn back to yellow. Furthermore, annealing of the yellow sample can again lead to the formation of the perovskite, indicating that the reaction between PbCl_2_ and CH_3_NH_3_I is reversible. In this case, the reaction depicted by (1) can be more precisely described by equation (3):


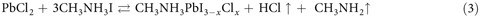


Since ΔG = ΔH − T ·ΔS (ΔG is the Gibbs free energy, ΔH is the enthalpy difference and ΔS is the entropy difference), the reduction of Gibbs free energy induced by the entropy increase becomes increasingly significant as the temperature increases. Apparently, the release of HCl and CH_3_NH_2_ favours the entropy increase and high temperature promotes the forward reaction.

To explore the reaction pathways, we apply *in situ* XRD to probe the crystal structure evolution–associated intermediate chemical reactions. The precursor film was firstly pre-dried at 60 ^o^C. XRD measurement displays several sets of diffraction patterns (Fig. 2a). First, the diffraction peak at 11.99^o^ can be ascribed to PbI_2_^20^. However, we did not observe diffraction peak belonging to PbCl_2_. These two results indicate that an ion exchange between Cl^-^ in PbCl_2_ and I^-^ in CH_3_NH_3_I has occurred, described as reaction (4).


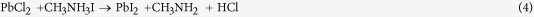


Second, the peaks at 15.53^o^ and 31.37^o^ can be assigned to the diffractions of CH_3_NH_3_PbCl_3_,^22^ suggesting that the reaction between PbCl_2_ and CH_3_NH_3_Cl has also happened along with the ion exchange as in reaction (5). There is also XRD diffraction peak at 14.02^o^ corresponding to CH_3_NH_3_PbI_3_ observed at this stage, indicating the reaction (6).


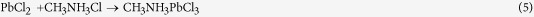



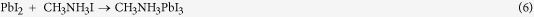


The diffractions at 11.38^o^, 16.64^o^, 28.42^o^ and 18.03^o^ cannot be assigned to any perovskite or lead halide; these peaks are presumably associated with the complex intermediate phase that composed of Pb halide and the organic species (Pb-complex)^23^.

At the beginning of heating at 100 ^o^C, the typical XRD diffraction pattern (Fig. 2a) of a perovskite at 14.02^o^, 28.27^o^, 31.75^o^ and 43.06^o^ becomes more pronounced, which is ascribed to the (100), (200), (210) and (300) plane with a cubic structure^18^. Prolonged heating leads to a gradual disappearance of the peaks of PbI_2_ and CH_3_NH_3_PbCl_3_, accompanied by the growth of the diffraction peaks of CH_3_NH_3_PbI_3_ (Fig. 2a,b), indicating that reaction (7) is also occurred. In this regard, the formation of CH_3_NH_3_PbCl_3_ at the initial stage is a kinetically favourable process when there are relatively higher concentrations of PbCl_2_ and CH_3_NH_3_Cl, while the formation of CH_3_NH_3_PbI_3_ is a thermodynamically favourable reaction^24^,^25^.


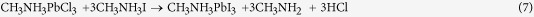


Another notable observation from the XRD pattern is that the (100) and (200) diffraction peaks downshift continuously during the annealing at 100 ^o^C (Fig. 2b,c), which corresponds to the lattice constant increasing from 0.6300 nm to 0.6315 nm from the initial annealing at 100 ^o^C till the end of the reaction. The ionic radius of Cl^−^ and I^−^ are 167 and 206 pm, so the increase in lattice parameter is ascribed to the replacement of Cl^−^ by I^−^ in the hybrid perovskite, through reaction (7) and (8) as indicated in Fig. 2d. In addition, the weak diffractions associated with Pb-complex at 11.38^o^, 16.64^o^, and 18.03^o^ gradually disappears as a result of the consumption of Pb for the formation of perovskite at elevated temperature (Fig. 2a).


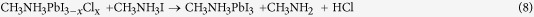


The kinetic factor that influences the reaction rate is usually reflected by the activation energy (*E*_a_) of the reaction. To obtain *E*_a_, we first of all explore a parameter that can quantify the reaction rate. Considering the nature of the solid-state reaction, the gas release speed is associated with the reaction rate. If we assume that the overall reaction is described by reaction (9) below, a complete transformation to the perovskite leads to a weight loss of 17.9% as the gaseous products escape. This complete transformation to a pure-iodide based perovskite is reasonable since prolonged heating often results in undetectable chlorine content, both observed in our investigations and in the literature^20^.


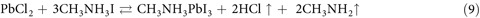


**Figure 3:**
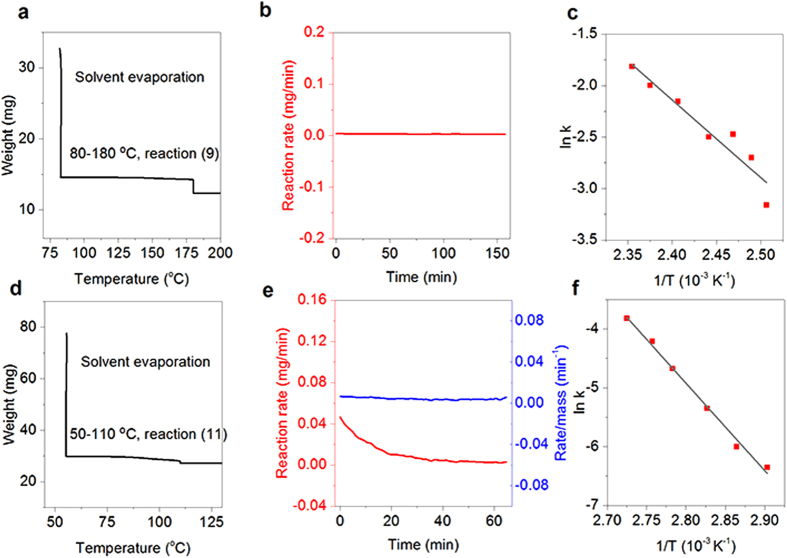
Thermogravimetric analysis on the PbCl_2_ + 3CH_3_NH_3_I reaction system. **a**, The weight loss of the reaction precursors consists of PbCl_2_, CH_3_NH_3_I and DMF at 80 ^o^C to evaporate the solvent, and 80–180 ^o^C to initiate the reaction. **b**, Reaction rate–time plot of the PbCl_2_ + 3CH_3_NH_3_I reaction system at 150 ^o^C. **c**, ln*k*–1/*T* plot of the reaction. **d**, The weight loss of the PbI_2_ + CH_3_NH_3_I system at 60 ^o^C and 60–110 ^o^C. **e**, Reaction rate–time plot of the reaction towards CH_3_NH_3_PbI_3_. **f**, ln*k*–1/*T* plot of the PbI_2_ + CH_3_NH_3_I system.

In the thermogravimetric analysis (TGA) of reaction (9), the precursor solution was pre-dried at 80 ^o^C for 2.5 hours to evaporate the DMF out of the mixture (Fig. 3a and Supplementary Fig. 3). Subsequently, the precursor powder was heated to initiate the reaction. It should be noted that the amount of the reaction precursor for TGA measurement (milligram scale) is significantly greater than that for device fabrication, the latter of which is usually a thin layer of film of several hundred nanometers thick. Therefore, the required heating temperature for the reaction in the TGA measurement must be higher than the latter due to the temperature gradient inside the precursors. We thus recorded the reaction rate in the temperature ranging from 80 to 180 ^o^C. We found that toward the end, the weight loss fraction reaches 15.2% (Fig. 3a), which is a little smaller than the theoretical weight loss fraction, possibly because the reaction has occurred during the pre-drying process. However, this would not influence the rationality of our method in that we quantify the gas release associated reaction rate at a specific temperature and duration.

We thus plot the reaction rate (*R*_*r*_) against time (*t*) to determine the order of the reaction. The constant reaction rate as a function of time (Fig. 3b) indicates that reaction (9) is a zero-order reaction. Hence, *R*_*r*_ = *k* (*k* is the rate constant of the forward reaction). The natural logarithm form of Arrhenius equation can be expressed by formula (10).


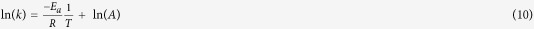


where *A* is the pre-exponential factor, *E*_a_ is the activation energy, and *R* is the universal gas constant. Therefore, from the ln(*k*) *−*1/*T* plot (Fig. 3c) we can calculate the value of −*E*_a_/*R* from the slope. The value of *E*_a_ is then calculated to be 69 kJ mol^−1^. Generally, *E*_a_ value of a chemical reaction is in the 40–400 kJ mol^−1^ range. The small *E*_a_ explains the rapid reaction between PbCl_2_ and CH_3_NH_3_I even at a mild condition. Since the reactions (4), (5) and (6) are nearly instantaneously occurred after drying at 60 ^o^C. The calculated *E*_a_ represents required energy for the conversion of Pb-complex, CH_3_NH_3_PbCl_3_ (reaction d), CH_3_NH_3_PbI_3−x_Cl_x_ (reaction e), to CH_3_NH_3_PbI_3_.

Since the conversion towards final all-iodide perovskite is reversible and involves gaseous products generation, the increase in reactant would increase the distance for the gases diffusion out of the film. Furthermore, the increased diffusion length would increase the back reaction. An overall result is that the reaction rate did not show increment along with the increase of reactant amount. This is the reason why the formation of CH_3_NH_3_PbI_3_ displays zero-order reaction characteristics.

Different from the perovskite synthesis using PbCl_2_ and CH_3_NH_3_I as the precursors, the reaction between PbI_2_ and CH_3_NH_3_I is conducted in a 1:1 ratio to generate CH_3_NH_3_PbI_3_ without chlorine doping (denoted as “PbI_2_ + CH_3_NH_3_I system”). The electronic properties of the two kinds of perovskites and their film formability are quite different^26^,^27^, which in turn requires different device architecture for efficient energy conversion^17^. Here we focus only on the chemical perspective of the reaction. To quantify the reaction rate, we monitor the DMF releasing speed since a number of DMF molecules are coordinated with PbI_2_^28^. We conducted the TGA experiment to determine the coordination ratio. After sufficiently pre-drying the perovskite precursor solution containing PbI_2_ and CH_3_NH_3_I at 60 ^o^C, the yielded light yellow powder was heated at elevated temperature to facilitate the reaction. The weight loss of the reaction was found to be 10.2% (Fig. 3d). From theoretical calculation, a one-to-one coordination would result in the weight percentage of DMF being 10.5% according to reaction (11). Therefore, the weight loss due to the escape of DMF can be used to evaluate the reaction rate.


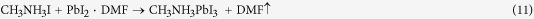


We then plot the reaction rate *R*_*r*_ (at constant temperature of 110 ^o^C) against *t* to determine the order of the reaction (Fig. 3e). The plot fits well with a first order reaction. The *E*_a_ value is calculated to be 110 kJ mol^−1^ according to the ln(*k*) – 1/*T* plot (Fig. 3f), which is considerably higher than that of PbCl_2_ + 3CH_3_NH_3_I system (69 kJ mol^−1^). For the analysis of *E*_a_, we characterized the dried mixtures of the two systems. It was found that the “PbCl_2_ + 3CH_3_NH_3_I” system forms poor crystallized product while the “PbI_2_ + CH_3_NH_3_I” system forms better crystallized material according the XRD characterization (Supplementary Fig. 4). Presumably, the barrier for the transformation from poor crystallized (amorphous) form to the perovskite is lower than that from well crystallized substance to the perovskite. This is the reason why the “PbCl_2_ + 3CH_3_NH_3_I” system possesses low *E*_a_ when compared with that of “PbI_2_ + CH_3_NH_3_I” system.

However, in the synthesis, the reaction speed of “PbI_2_ + CH_3_NH_3_I” system is much faster than that of “PbCl_2_ + 3CH_3_NH_3_I” system. To understand this issue, we consider *A* in the Arrhenius equation, which is the parameter to evaluate the effective collision in the chemical reaction. They are 9 × 10^15^ and 6 × 10^6^ respectively for the “PbI_2_ + CH_3_NH_3_I” and “PbCl_2_ + 3CH_3_NH_3_I” systems according to Fig. 3c,f. The overall reaction speed is dependent on both *A* and *E*_a_ at a specific temperature on the ground of Arrhenius equation; this explains why the higher *E*_a_ in “PbI_2_ + CH_3_NH_3_I” system can still possess a faster reaction speed than “PbCl_2_ + 3CH_3_NH_3_I” system.

**Figure 4:**
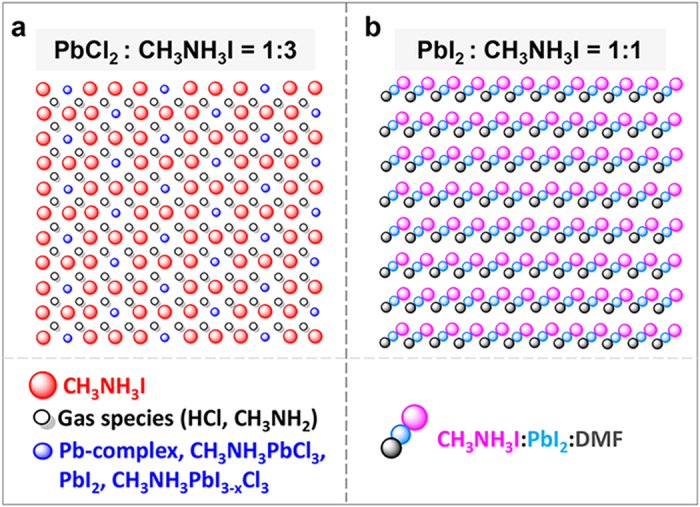
Schematic illustration of the distribution of precursor materials in the two reaction systems. **a**, the “PbCl_2_ + 3CH_3_NH_3_I” system dried at 60 ^o^C for 2 hrs. **b**, the “PbI_2_ + CH_3_NH_3_I” system dried at 60 ^o^C for 2 hrs.

The “collision” probability is analyzed in Fig. 4. In the “PbCl_2_ + 3CH_3_NH_3_I” system, the gaseous species such as CH_3_NH_2_ and HCl would block the diffusion of the reactants (Fig. 4a), i.e. PbI_2_, Pb-complex, CH_3_NH_3_PbCl_3_, CH_3_NH_3_PbI_3-x_Cl_x_, and CH_3_NH_3_I, which retard the reaction speed. In the “PbI_2_ + CH_3_NH_3_I” system, according to the TGA result, the final molar ratio of the reactants is 1:1:1 of CH_3_NH_3_I:PbI_2_:DMF. The XRD characterization show crystallized structure (Supplementary Fig. 4). The diffraction pattern cannot be assigned to any perovskite or the precursor material. It is most likely a complex intermediate of CH_3_NH_3_I∙PbI_2_∙DMF. In this case, the following reaction mechanism is possible.


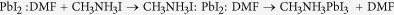


Since the complex is composed of reactants of CH_3_NH_3_I and PbI_2_, the reaction for the formation of CH_3_NH_3_PbI_3_ doesn’t require significant mass diffusion (Fig. 4b). The reaction speed is dependent on the concentration of the intermediate state, showing first-order reaction feature, where reaction rate 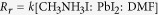. The perovskite film synthesized by the reaction between PbI_2_ and CH_3_NH_3_I is featured as poor film formability as discussed in the introduction. The porous film leaves many channels for the DMF escape. Therefore, the release of DMF is not a rate determining step, in contrast to the “PbCl_2_ + 3CH_3_NH_3_I” system where the uniform and compact film could retain a high concentration of gaseous species resisting the forward reaction.

**Figure 5:**
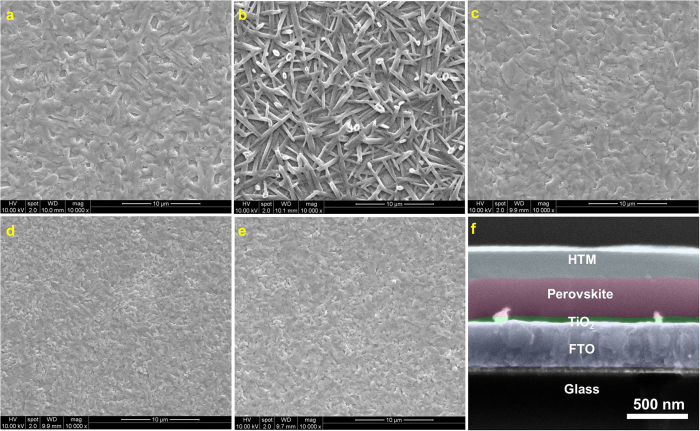
Morphological characterization of the active layer and device cross section. **a**, SEM image of the active layer prepared by the precursor composed of PbCl_2_ and CH_3_NH_3_I with 1:3 molar ratio; **b**, SEM image of the active layer prepared by the precursor composed of PbI_2_ and CH_3_NH_3_I with 1:1 molar ratio; **c**, **d**, and **e**, SEM images of the photoactive layer prepared using mixed precursors of CH_3_NH_3_I, PbCl_2_ and PbI_2_, the molar ratios are of 1.5:0.25:0.75, 2.0:0.5:0.5 and 2.5:0.75:0.25, respectively. **e**, SEM image of a typical cross section of a complete planar heterojunction device.

Slow reaction kinetics is usually beneficial to the formation of a uniform perovskite film. Thus the PbCl_2_ + 3CH_3_NH_3_I system can produce uniform film with crystal sizes of around 800 nm (Fig. 5a). The crystal domain size is estimated based on the SEM images in Fig. 5a. The rapid reaction in PbI_2_ + CH_3_NH_3_I system usually gives rise to crystal domain sizes less than 200 nm with poor surface coverage (Fig. 5b). Generally, large grain size would cause internal mechanical strain and dewetting of the film on the substrate (Supplementary Fig. 6). The opposite aspect could increase the grain boundary that impedes the carrier transport. Both of the extremes are harmful for the device performance. Appropriate reaction kinetics is required for a high quality film^29^. Therefore, the use of various additives during reaction or solvent annealing after film formation has been developed for the high quality films^13^,^30^,^31^,^32^. Herein, we combine the two reaction kinetics together to find an optimal crystallinity. A series of mixtures of CH_3_NH_3_I, PbCl_2_ and PbI_2_ with molar ratios of 1.5:0.25:0.75, 2.0:0.5:0.5, 2.5:0.75:0.25 were prepared. The required annealing durations are 15, 23, 33 min and the average crystal sizes were measured to be 350 nm, 460 nm and 650 nm, respectively. All of the films display uniform morphologies with high surface coverage (Fig. 5c-e). These films were used as the absorber layers for the solar cells, in which 2,2′,7,7′-tetrakis-(N,N-di-p-methoxyphenylamine)9,9′-spirobifluorene (spiro-OMeTAD) was utilized as the hole transporting material (HTM) and evaporated silver was applied as the metal contact. A cross-section of a typical device is shown in Fig. 5f, where the thicknesses of TiO_2_ compact layer, perovskite, HTM and Ag are 50, 350, 200 and 100 nm, respectively.

**Figure 6:**
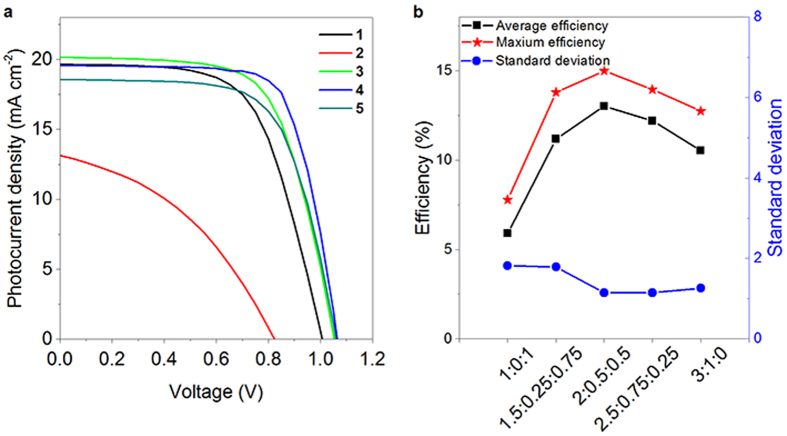
Photovoltaic property of the devices based on different film preparation method. **a**, Current density-voltage response of device **1** to **5** measured under AM 1.5 G, one sun illumination. The precursors for the photoactive layer of device **1** to **5** contain CH_3_NH_3_I, PbCl_2_ and PbI_2_ with molar ratios of 3:1:0, 1:0:1, 1.5:0.25:0.75, 2.0:0.5:0.5, 2.5:0.75:0.25. **b**, The plots of average and highest efficiency values and standard errors based on 20 devices.

The photocurrent density-voltage responses of the devices (Fig. 6a) and the calculated parameters are summarized in Table 1. When PbCl_2_ and CH_3_NH_3_I are used as the precursors (device **1**), the PCE can reach 12.26%, with a short-circuit current density (*J*_sc_) of 19.66 mA cm^−2^, an open-circuit voltage (*V*_oc_) of 1.00 V, and a fill factor (FF) of 0.62. The average PCEs and standard deviations based on over twenty devices of each set are summarized in Fig. 6b. The perovskite film fabricated with PbI_2_ and CH_3_NH_3_I as the precursors (device **2**) leads to a PCE of 4.29%, with *J*_sc_ of 13.14 mA cm^−2^, *V*_oc_ of 0.82 *V* and *FF* of 0.40. When using the mixture of lead precursors with CH_3_NH_3_I:PbCl_2_:PbI_2_ of 1.5:0.25:0.75 (device **3**), 2.0:0.5:0.5 (device **4**) and 2.5:0.75:0.25 (device **5**), the PCEs are 13.78%, 15.00% and 13.05%, respectively. In the device fabrication, we found that to maintain 1:3 ratio between PbCl_2_ and CH_3_NH_3_I and 1:1 ratio between PbI_2_ and CH_3_NH_3_I in the precursor is crucial for the high–quality photoactive films.

Remarkably, the FFs of the three devices (**3**, **4**, **5**) with films prepared by using mixed PbCl_2_ and PbI_2_ sources are larger than that of the two devices fabricated using only PbCl_2_ or PbI_2_ as the lead precursor. The FF for the optimal device reaches 0.72 (device **4**). FF is a parameter that reflects the quality of the device and is associated with the total resistance of the devices. The FFs of the devices based on the planar heterojunction (Fig. 5f) are usually smaller than 0.70^15^,^33^. The optimizations on other components such as the TiO_2_ compact layer^34^, the band gap of TiO_2_ and work function of ITO, could improve the FF to a value higher than 0.70^3^. Herein, we obtain high FF by meticulously engineering the crystallinity of the perovskite. By balancing the two kinetics, i.e. the slow PbCl_2_ + 3CH_3_NH_3_I reaction system provides relaxation time for generating uniform and dense film with a high degree of crystallization while the fast kinetics PbI_2_ + CH_3_NH_3_I is able to reduce the crystal size for minimizing the internal mechanical strain and dewetting, optimized crystal size and film formability can be achieved for enhanced carrier transport property and high FF.

## Conclusion

In conclusion, we have established the chemical equations for the synthesis of organolead trihalide perovskite by the two approaches. Both of the two types of reactions involve the generation of gas species, this is thermodynamically favourable for the reaction especially at elevated temperatures. The kinetic reasons for the rapid reaction are identified; the activation energies are calculated to be 69 and 110 kJ mol^−1^ for the PbCl_2_ + 3CH_3_NH_3_I and PbI_2_ + CH_3_NH_3_I reaction systems. In addition to the activation energies required for the reactions, we also found that, in the PbI_2_ + CH_3_NH_3_I system, the high degree of “collision” contributes to the rapid reaction. However, in the PbCl_2_ + 3CH_3_NH_3_I system, the removal of the side products is a key factor that influences the reaction rate. Since nearly all the solution processed perovskite materials are through the two types of reactions, our investigation is expected to generate broad interests in the fundamental study of the perovskite synthesis. The engineering of crystallinity of the perovskite provides an effective methodology for the fabrication of high-quality devices and probing the working principles related to the crystallite size.

## Methods

### Material preparation

All materials were purchased from commercial suppliers and used as received unless stated otherwise. CH_3_NH_3_I was synthesized according to a literature method and dried in a vacuum oven at 60 ^o^C for 10 h before using^13^. A series of perovskite precursor solutions were prepared in anhydrous DMF with a molar ratio of CH_3_NH_3_I: PbCl_2_: PbI_2_ as 3:1:0 (none of PbI_2_, for device 1), 1:0:1 (none of PbCl_2_, for device 2), 1.5:0.25:0.75 (device 3), 2:0.5:0.5 (device 4) and 2.5:0.75:0.25 (device 5). The total concentration of lead salt in each solution was kept at 0.88 M.

### Device Fabrication

FTO-coated glass with sheet resistance of 14 Ω sq^−1^ was washed by sonication with deionized water, ethanol and acetone and then treated with oxygen plasma for two minutes. A compact layer of TiO_2_ was deposited on the FTO substrate by spin-coating the titanium precursor (0.24 M titanium isopropoxide and 0.12 M HCl in ethanol) at 5000 r.p.m. for 60 s following by calcination on a hotplate at 500 ^o^C for 40 min. Subsequently, the perovskite solution was spin-coated on the cooled TiO_2_/FTO substrate in a nitrogen-filled glovebox at 3000 r.p.m. for 60 s. It was annealed on a hotplate at 100 ^o^C for the reaction and crystallization of the perovskite. The optimized annealing time for the above-mentioned five precursors is 45 min, 10 min, 15 min, 23 min and 33 min, respectively. Then, the HTM, spiro-OMeTAD, was deposited by spin coating a solution (72.5 mg sprio-OMeTAD, 42.7 μL 4-tert-butylpyridine (tBP) and 26.3 μL lithium-bis(trifluoromethanesulfonyl)imide (Li-TSFI) stock solution (520 mg mL-1 in acetonitrile) in 1 mL chlorobenzene) at 5000 r.p.m. for 60 s. After oxidizing the HTM layer in air for 15 h, the cell was completed by thermally evaporating a 100 nm-thick silver layer.

### Materials Characterizations

The TGA was performed in nitrogen atmosphere at a flow rate of 20 mL min^−1^. 25 μL perovksite solution containing CH_3_NH_3_I and PbCl_2_ of a molar concentration of 2.64 M and 0.88 M (mass percentage 42%) was held at 80 ^o^C in a ceramic crucible for 150 min until the weight kept unchanged. A weight loss of 56% meant that DMF nearly totally evaporated. Then the remaining solid was heated from 80 ^o^C to 180 ^o^C at a rate of 5 ^o^C min^−1^ during which CH_3_NH_3_PbI_3-*x*_Cl_*x*_ formed in the reaction (Supplementary Fig. 3). In another experiment, the system was heated up to 150 ^o^C and held at this temperature for 150 min in order to monitor the reaction rate against time at isothermal condition.

For the perovskite solution containing CH_3_NH_3_I and PbI_2_ of a molar ratio of 1:1, 65 μL of the 36% (mass percentage) solution was held at 60 ^o^C until the solvent DMF was totally evaporated. Mass loss of 60% meant that one DMF molecule is possibly binding with one Pb atom in the remaining solid. Then the yielded light yellow solid was heated from 60 ^o^C to 110 ^o^C and held at 110 ^o^C till the complete reaction (Supplementary Fig. 3).

The released gas during the reaction of the perovskite precursor was collected and analyzed by FTIR and XRD. For the FTIR analysis, 2 mL perovskite solution containing CH_3_NH_3_I and PbCl_2_ of a molar ratio of 3:1 was dried in vacuum at 80 ^o^C to evaporate the solvent which yielded light yellow solid. This yellow solid was then heated in an Erlenmeyer flask at 150 ^o^C to facilitate the chemical reaction and the released gas was purged through NaOH or P_2_O_5_ powder to collect the basic or acidic gas using a syringe. The collected gas was analyzed by FTIR in a ZnSe-window gas cell between the wavenumber of 500 cm^−1^ and 3500 cm^-1^ with a step of 0.5 cm^−1^. The released substance during the annealing of the perovskite precursor film was also collected using a glass substrate for the XRD characterization. Typically, the perovskite solution was spin-coated on an FTO-coated glass at 2000 r.p.m for 60 s and annealed at 100 ^o^C on a hotplate during which a piece of glass was covered on top of the film and separated about 100 μm above it. White substance was noticed to deposit on the covering glass. For collecting enough material for XRD characterization, this covered-annealing process was repeated for five times with each time lasting for 50 min.

For the *in situ* XRD characterization of the annealing perovskite film (PbCl_2_ + 3CH_3_NH_3_Cl system), the readily spin-coated perovskite film on TiO_2_/FTO substrate was transferred from a glovebox into an XRD characterization cell which was filled with nitrogen. The temperature of the characterization cell was held at 60 ^o^C at first and then increased to 100 ^o^C and kept for 50 min until the complete formation of CH_3_NH_3_PbI_3_. During this annealing stage, *in situ* XRD characterization (Cu K_α_, λ = 1.5406 Å) was performed from 10 degree to 50 degree (2θ) at a speed of 5 degree/min at a condition of 40 kV and 80 mA. The characterization process was repeated every ten minutes to monitor the phase change during the annealing stage.

For other XRD characterizations, the annealed perovskite films were tested in ambient condition at a speed of 10 degree min^−1^ from 10 degree to 70 degree (2θ) (Supplementary Fig. 5).

### Device Characterizations

The J-V characterization of the solar cells was conducted in a nitrogen-filled glovebox under the luminescence of AM 1.5 G solar-simulated light with an intensity of 100 mW cm^−1^. Two probes mounted on the micropositioners were put in touch with the FTO and the silver electrodes, respectively. A scanning voltage from −0.2 V to 1.2 V was applied across the two electrodes at a step of 0.05 V and the corresponding current was recorded. All the cells had an active area of 0.12 cm^2^ defined by the silver electrode.

The incident photon current efficiency (IPCE) of the cells was characterized in air under the luminescence of monochromatic light from 300 nm to 800 nm in a DC mode without bias light. The output current of the cell was record at each wavelength. With the incident light powder known, the conversion efficiency from incident photons to the output charges was thus calculated for the whole spectrum.

### Calculation of Ea

The reaction rate (*R*_*r*_) of the PbCl_2_ + 3CH_3_NH_3_I system was derived by taking a derivative on the weight of the reactant as a function of time at an isothermal temperature of 150 ^o^C. The calculated reaction rate was 0.004 mg min^−1^ and gradually decreased to 0.003 mg min^−1^ after 150 min which could be considered as constant. It should be noted that the amount of the reactant is significantly larger than that in the device fabrication, so the required time for completing the reaction becomes much longer. On the other hand, with the generation of product in a solid-state reaction, the diffusion time of reactants becomes longer and longer, the apparent reaction rate thus becomes slower and slower.

The reaction rate of the PbI_2_ + CH_3_NH_3_I system was also calculated in a similar way at an isothermal temperature of 110 ^o^C over a timescale of 60 min. The initial reaction rate was calculated to be 0.047 mg min^−1^ and continuously decreased to 0.003 mg min^−1^. When dividing the reaction rate by the weight of the reactant, we got a constant value about 0.006 min^−1^ which means that the reaction rate is proportional to the amount of reactant and this is a first-order reaction.

The activation energy of the above two reaction systems was calculated from the fitted slope of ln(*k*) – 1/*T* plot (*k* represents the reaction rate constant and *T* is thermodynamic temperature). Since the reaction between CH_3_NH_3_I and PbCl_2_ is a zero order one, then we can write 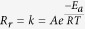 (*A* is the pre-exponential factor and *R* is the universal gas constant) based on Arrhenius equation. The reaction rate was calculated within a temperature range from 120 ^o^C to 150 ^o^C. For the reaction of the PbI_2_ + CH_3_NH_3_I system, we can write that 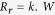 where *W* respresents the weight of the reactant since this is a first-order reaction. The data for plotting ln(*k*) – 1/*T* is in the temperature range between 70 ^o^C and 100 ^o^C.

## Additional Information

**How to cite this article**: Wang, B. *et al*. Elucidating the Reaction Pathways in the Synthesis of Organolead Trihalide Perovskite for High-Performance Solar Cells. *Sci. Rep.* **5**, 10557; doi: 10.1038/srep10557 (2015).

## Supplementary Material

Supplementary Information

## Figures and Tables

**Table 1:** Perovskite film fabrication and photovoltaic parameter of the devices.

**Device**	**CH**_**3**_**NH**_**3**_**I:PbCl**_**2**_**:PbI**_**2**_**(molar ratio)**	**Annealing time (min)**	***V***_**oc**_ **(V)**	***J***_**sc**_ **(mA cm**^**−2**^)	**FF**	**PCE (%)**
**1**	3:1:0	45	1.00	19.66	0.62	12.26
**2**	1:0:1	10	0.82	13.14	0.40	4.29
**3**	1.5:0.25:0.75	15	1.05	20.18	0.65	13.78
**4**	2.0:0.5:0.5	23	1.06	19.58	0.72	15.00
**5**	2.5:0.75:0.25	33	1.06	18.58	0.66	13.05

The molar ratio of CH_3_NH_3_I:PbCl_2_:PbI_2_ in the fabrication of perovskite film, optimized annealing time, *V*_oc_, *J*_sc_, FF and PCE of device 1 to 5. The PCE was measured under standard AM 1.5 G illumination (1,000 W m^−2^).
