# Supplementary material for: Elucidating the Reaction Pathways in the Synthesis of Organolead Trihalide Perovskite for High-Performance Solar Cells
Source: Sci Rep. 2015 May 28;5:10557. doi: 10.1038/srep10557 (PMC4447066; doi:10.1038/srep10557)
Supplement: Supplementary Information [file srep10557-s1.pdf]

Supplementary Information

Elucidating the Reaction Pathways in the Synthesis of Organolead Trihalide Perovskite for High-Performance Solar Cells

Baohua Wang, King Young Wong, Xudong Xiao and Tao Chen*

Department of Physics, The Chinese University of Hong Kong, Shatin, N. T., Hong Kong, China

All correspondence should be addressed to: taochen@phy.cuhk.edu.hk (T. C.)

Supplementary Table 1: EDX results showing the atomic ratio of chlorine and iodine in the perovskite precursor film annealing for different time at 100 ^{o}C prepared using the precursor containing a 3:1 molar ratio of CH_3NH_3I and $PbCl_2$.

Annealing time (min)	Atom% Cl	Atom% I	Atomic ratio I/Cl
0	15.0	23.8	1.6
1	13.4	26.9	2.0
15	9.5	30.3	3.2
50	1.4	36.2	25.9

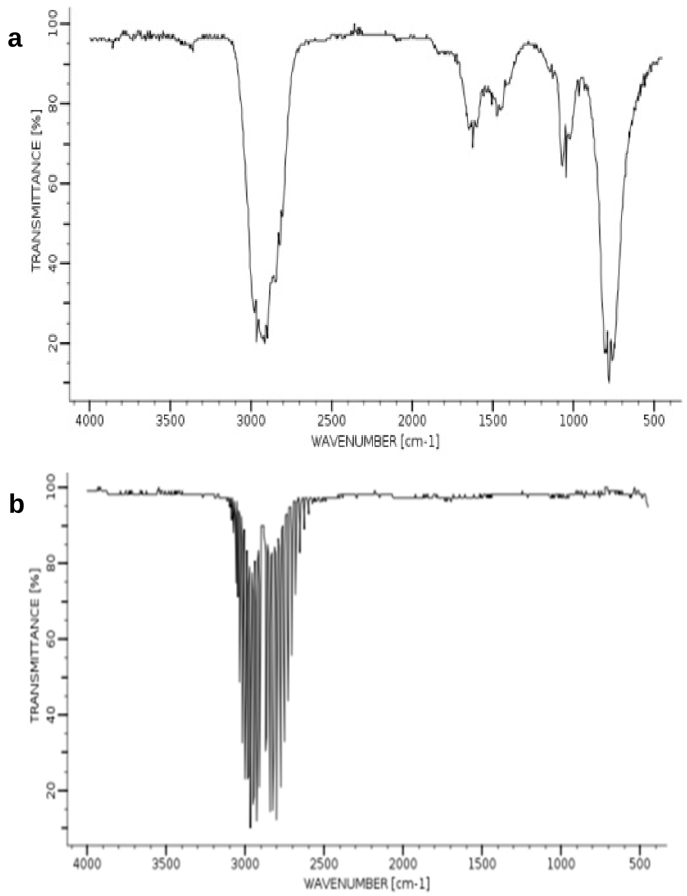

Supplementary Figure 1: **a**, Standard FTIR spectrum of CH_3NH_2 (spectrum ID BR136296). **b**, Standard FTIR spectrum of HCl (spectrum ID BR145446) from database of SciFinder.

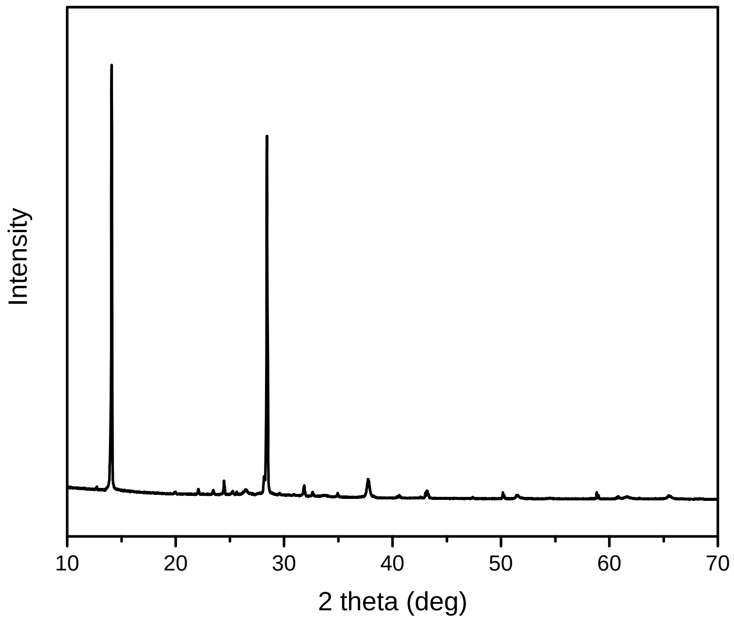

Supplementary Figure 2: XRD pattern of the perovksite film prepared from annealing the $PbCl_2$+$3CH_3NH_3I$ precursor in NH_3-containing atmosphere at 100 ^oC for 2 min.

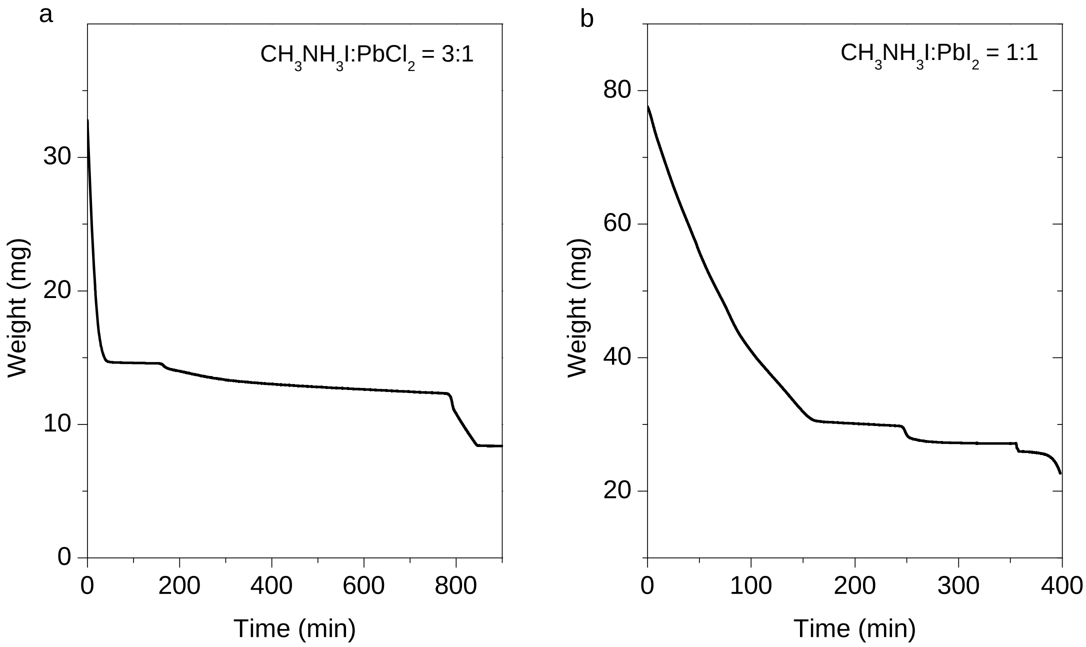

Supplementary Figure 3: TGA spectrum showing the weight of the perovskite precursor solution as a function of time. **a**, The temperature of the precursor solution containing CH_3NH_3I and $PbCl_2$ is held at 80 ^oC for 150 min and then increased to 180 ^oC and held at 180 ^oC for 600 min and further increase to 300 ^oC. **b**, The temperature of the precursor solution containing CH_3NH_3I and PbI_2 is held at 60 ^oC for 240 min and then increased to 110 ^oC and held further for 100 min and finally increased to 350 ^oC.

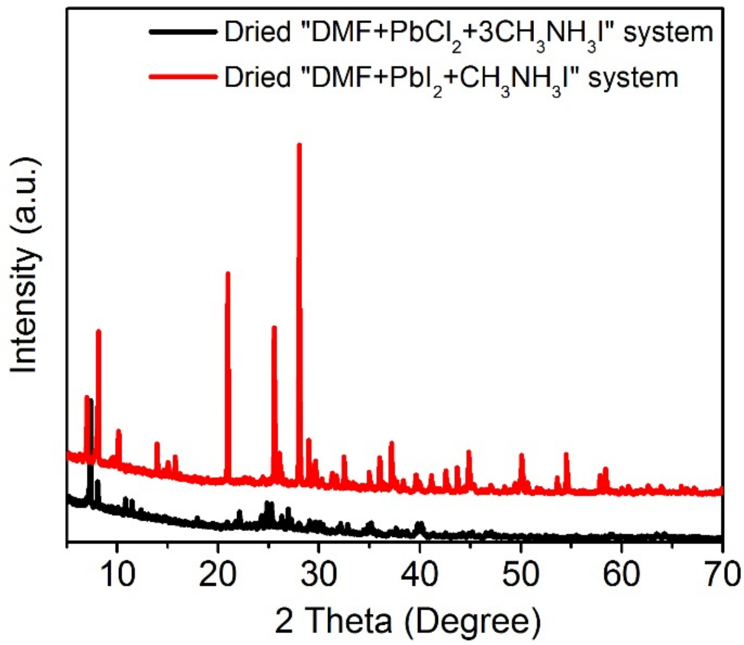

Supplementary Figure 4: XRD patterns of the solids got from vacuum-drying the solvent out of the $PbCl_2$+3CH_3NH_3I system and PbI_2+CH_3NH_3I system

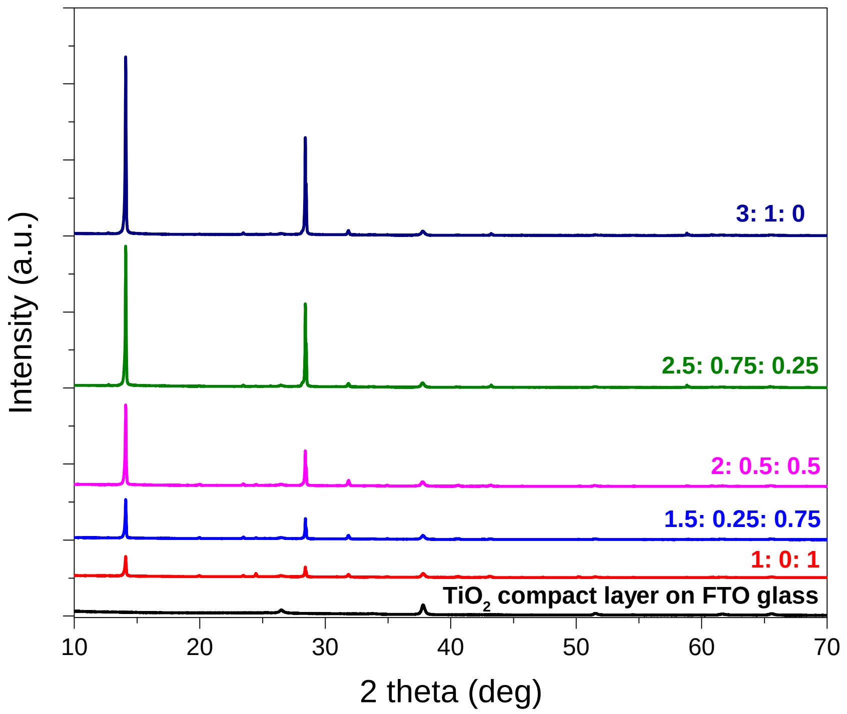

Supplementary Figure 5: XRD spectrum of the TiO_2/FTO substrate and the five perovskite film prepared using precursor solutions containing CH_3NH_3I, $PbCl_2$ and PbI_2 with molar ratios of 1:0:1(none of $PbCl_2$), 1.5:0.25:0.75, 2.0:0.5:0.5, 2.5:0.75:0.25 and 3:1:0 (none of PbI_2) on the substrate.

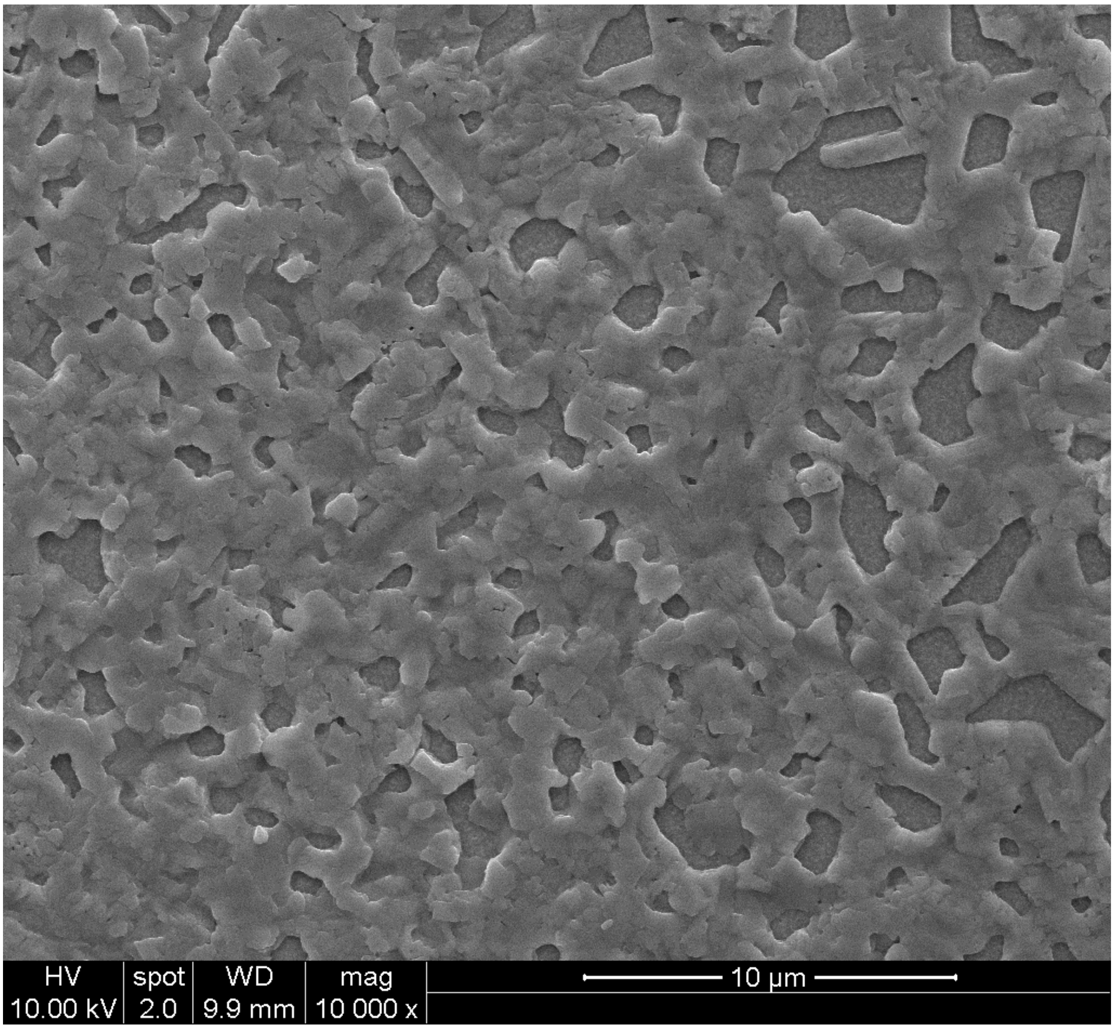

Supplementary Figure 6: SEM image of the perovskite film on a TiO_2/FTO substrate, which is prepared using the $PbCl_2+3CH_3NH_3I$ system. During annealing at 100 ^oC, a covering glass is placed on the top of the sample to retard the gas release. 10 hours is thus required for the complete reaction towards $CH_3NH_3PbI_{3-x}Cl_x$. The slow reaction can increase the size of crystallites to micron scale, but the surface coverage becomes very low.

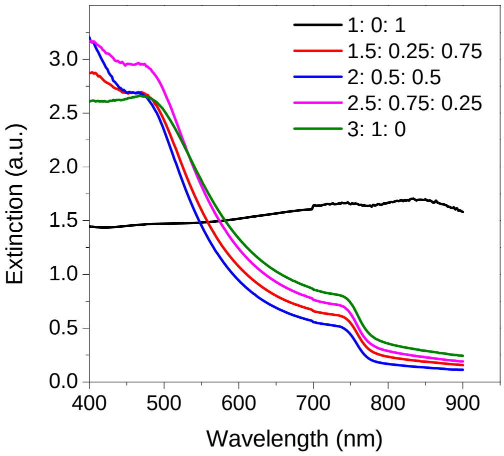

Supplementary Figure 7: UV-vis spectra of the perovskite films on TiO_2/FTO substrates prepared using different precursor solutions containing CH_3NH_3I, $PbCl_2$ and PbI_2 with a molar ratio of 1:0:1(none of $PbCl_2$), 3:1:0 (none of PbI_2), 1.5:0.25:0.75, 2.0:0.5:0.5 and 2.5:0.75:0.25, for device **1** to **5**, respectively.

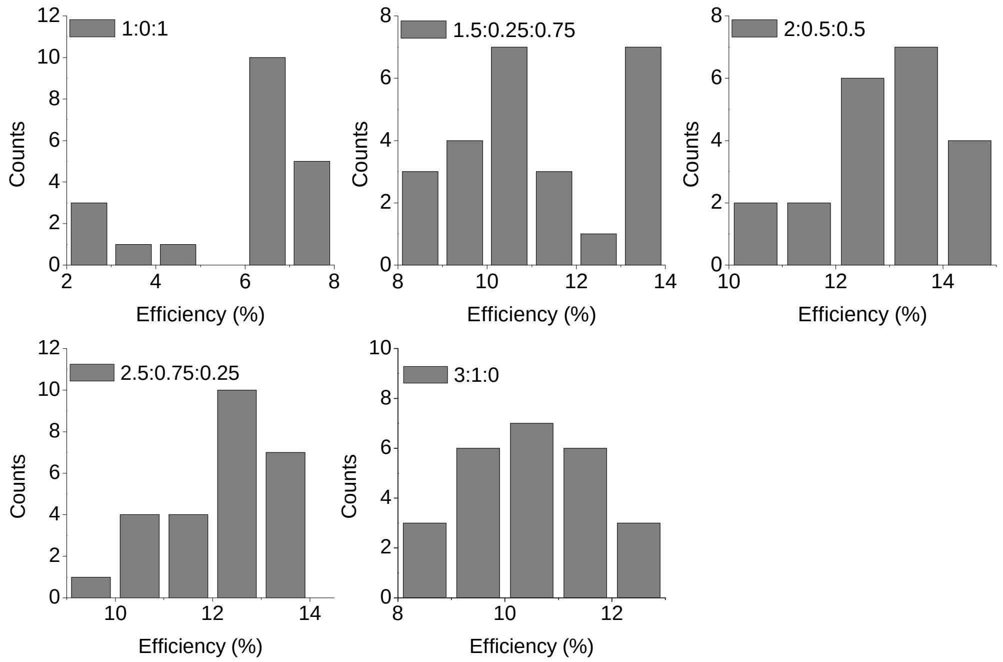

Supplementary Figure 8: Statistics of the PCEs of devices prepared using different precursor solutions containing CH_3NH_3I, $PbCl_2$ and PbI_2 of a molar ratio of 1:0:1(none of $PbCl_2$), 1.5:0.25:0.75, 2.0:0.5:0.5, 2.5:0.75:0.25 and 3:1:0 (none of PbI_2); the highest efficiency, average efficiencies and standard deviations are summarized in the main text in Fig. 5b.
